# Supplementary material for: An open-source framework for large-scale, flexible evaluation of biomedical text mining systems
Source: J Biomed Discov Collab. 2008 Jan 29;3:1. doi: 10.1186/1747-5333-3-1 (PMC2276192; doi:10.1186/1747-5333-3-1)
Supplement: Additional file 1 — Evaluation platform source code. The additional file contains the software evaluation framework discussed in this paper. The most current release can be downloaded from SourceForge.net [1]. See the accompanying README file for instructions on installation and use. Also included in the distribution is a collection of UIMA wrappers for some commonly used BioNLP tools and annotated corpora. [file 1747-5333-3-1-S1.zip › bionlp_uima_repository-1.0.1/docs/api/src-html/edu/uchsc/ccp/util/nlp/document/DocumentSectionTypes.html]

```
001    package edu.uchsc.ccp.util.nlp.document;
002    
003    
004    public interface DocumentSectionTypes {
005    
006            public static final String OTHER_NAME = "other";
007            public static final int OTHER_ID = 0;
008            
009            public static final String TITLE_NAME = "title";
010            public static final int TITLE_ID = 1;
011            
012            public static final String ABSTRACT_NAME = "abstract";
013            public static final int ABSTRACT_ID = 2;
014        
015        public static final int HW_TITLE = 20;
016        public static final int HW_ABSTRACT = 20;
017        
018        
019    }
```
